# Global Mapping of DNA Conformational Flexibility on *Saccharomyces cerevisiae*

**DOI:** 10.1371/journal.pcbi.1004136

**Published:** 2015-04-10

**Authors:** Giulia Menconi, Andrea Bedini, Roberto Barale, Isabella Sbrana

**Affiliations:** 1 Dip. Informatica, Università di Pisa, Largo Pontecorvo, Pisa, Italy; 2 Istituto Nazionale di Alta Matematica “Francesco Severi”, Piazzale Aldo Moro, Città Universitaria, Roma, Italy; 3 Dept. Mathematics and Statistics, The University of Melbourne Victoria, Australia; 4 Dip. Biologia, Università di Pisa, Via Derna, Pisa, Italy; Hellas, GREECE

## Abstract

In this study we provide the first comprehensive map of DNA conformational flexibility in *Saccharomyces cerevisiae* complete genome. Flexibility plays a key role in DNA supercoiling and DNA/protein binding, regulating DNA transcription, replication or repair. Specific interest in flexibility analysis concerns its relationship with human genome instability. Enrichment in flexible sequences has been detected in unstable regions of human genome defined fragile sites, where genes map and carry frequent deletions and rearrangements in cancer. Flexible sequences have been suggested to be the determinants of fragile gene proneness to breakage; however, their actual role and properties remain elusive. Our *in silico* analysis carried out genome-wide via the StabFlex algorithm, shows the conserved presence of highly flexible regions in budding yeast genome as well as in genomes of other Saccharomyces *sensu stricto* species. Flexibile peaks in *S. cerevisiae* identify 175 ORFs mapping on their 3’UTR, a region affecting mRNA translation, localization and stability. (TA)n repeats of different extension shape the central structure of peaks and co-localize with polyadenylation efficiency element (EE) signals. ORFs with flexible peaks share common features. Transcripts are characterized by decreased half-life: this is considered peculiar of genes involved in regulatory systems with high turnover; consistently, their function affects biological processes such as cell cycle regulation or stress response. Our findings support the functional importance of flexibility peaks, suggesting that the flexible sequence may be derived by an expansion of canonical TAYRTA polyadenylation efficiency element. The flexible (TA)n repeat amplification could be the outcome of an evolutionary neofunctionalization leading to a differential 3’-end processing and expression regulation in genes with peculiar function. Our study provides a new support to the functional role of flexibility in genomes and a strategy for its characterization inside human fragile sites.

## Author Summary

High DNA helix torsional flexibility characterizes sequences which are enriched in fragile sites, loci of peculiar chromosome instability inside human genome often associated with cancer genes. AT-rich flexible islands are suggested to be the determinants of chromosome fragility; however, the origin of their occurrence in cancer genes and the mechanism of chromosome breakage remain unknown. Here, we study DNA flexibility in budding yeast chromosomes. We found that flexibility is conserved in yeast species. Flexibile peaks identify 175 ORFs, mapping on their 3′-end untraslated region. (TA)n repeats of different extension shape the central structure of peaks and co-localize with polyadenylation signals. ORFs with peaks have decreased mRNA stability and prevalent regulatory functions. Our findings support the functional importance of flexibility peaks. They suggest that functional processes may be also at the origin of flexibility peaks presence inside cancer genes in human fragile sites. Definition of role of flexible sequences in genomes may help to understand the processes implied in cancer gene rearrangements.

## Introduction

DNA conformational flexibility is a function of the dsDNA sequence that defines how the molecule can bend or exhibit a torsion (twist motion) about its axis.

Flexibility is important in DNA supercoiling and shows particular significance in DNA-protein interaction. The relationship of flexibility with the nucleosome occupancy and DNA looping along the genomes determines its key role in many biological functions including the DNA regulation during transcription and replication and DNA repair [1].

The presence of areas of high DNA flexibility at the twist angle has been reported in several unstable regions of human genome, such as fragile sites. Fragile sites are regions peculiarly prone to DNA breakage, usually in conditions of replicational stress; the common fragile sites often map in association with genes involved in tumorigenesis, such as *FHIT*, *WWOX*; their instability causes cancer-specific recurrent deletion and translocation breakpoints [2]. While their molecular basis remains elusive, the identification in a number of them of AT-rich flexible islands, capable of forming stable secondary structures has suggested that flexible regions are good candidates for determinants of chromosome fragility [3, 4]. Effects on DNA stability through a structural interference with replication and a block of fork progression have been indicated as possible action mechanisms of flexible sequences [5]. Stalled forks and mitotic entry before replication completion have been indeed shown to be related to chromosome breakage in fragile regions [6]. New results, however, enlighten that also functional aspects are implied in chromosome fragility. Mapping of fragile sites in different cell type confirmed that their setting is tissue dependent and so epigenetically determined [7]. Consistently, fragile sites expressed in human lymphocytes show correlated breakage and are enriched in genes involved in immunity and inflammation, cell-type specific processes [8].

Experimental direct evidence for the role of flexibility in genomic instability has been obtained by using a genetic assay in yeast, where the insertion of a short AT-rich sequence that spans the peak of highest flexibility of the human fragile site *FRA16D* has been demonstrated to be able to increase chromosome breakage [9]. A support to this model comes from the observation in human genome that AT-rich flexibility peaks also lie at breakpoints of chromosome rearrangements involving the LCR22A-D region of 22q11.2 chromosome, a highly unstable segmental duplication implied in constitutional genomic diseases. [10].

In this paper we approach the problem of biological meaning of DNA helix flexibility by analysing budding yeast chromosome sequences. Yeast has a very compact genome which however comprises a large number of eukaryotic typical genomic elements. A very favourable condition is the large availability of genome-wide data concerning the structural and functional aspects. To this aim, we developed a computer program that predicts the flexibility of the DNA helix by measurements of the twist angle between consecutive base pairs, implementing the TwistFlex software previously developed [11] for the analysis of human fragile sites [3, 12] and its adaptation to fast long sequences analysis.

We present here a high resolution map of twist-angle deviation for the complete genome of *Saccharomyces cerevisiae* [13]. We determined the presence of 183 flexibility peaks. We defined peaks as segments of genome with twist flexibility above a fixed threshold (i.e. twice the standard deviation). We mapped the location of the flexibility peaks within the yeast genome using the SGD [14] and data reported in literature, both uploaded into the UCSC Genome Browser [15]. Flexibility peaks appear on the 3′UTR of 175 ORFs in *S. cerevisiae*, which share common features. The connection between flexibility peaks and ORFs could be the evolutionary outcome of modified canonical polyadenylation elements, leading to a differentiated 3′-end processing and gene expression regulation.

## Results

### Genomic distribution of flexibility peaks

**Fig 1:**
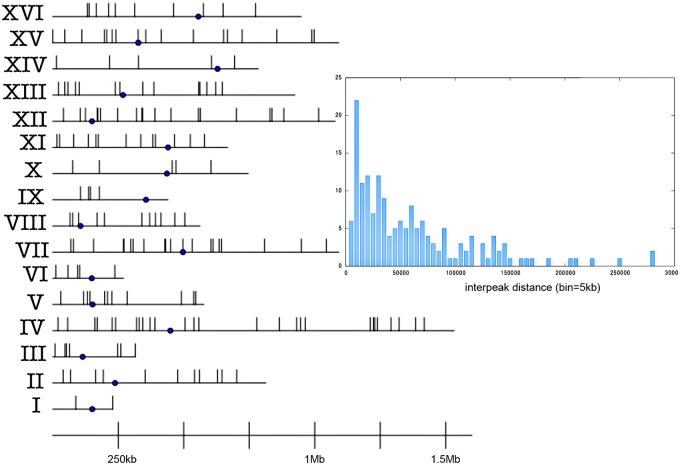
Chromosomal map of flexibility peaks. The point is the centromere. Inset: Distribution of distance between adjacent peaks in complete yeast genome (each bin spans 5kb and counts all the peaks within that distance to the nearest one).

The analysis of the first comprehensive map of twist flexibility values reveals the presence of 183 peaks which are 250*bp* long on average (longest 975*bp*, shortest 188*bp*). In the following, peaks shall be denoted by peakIV-16, meaning the 16th peak within chrIV. Their chromosomal map shows no enrichment at specific chromosome arms or at centromere or telomere positions/regions (Fig. 1). The longest chromosomes (chrIV, chrVII, chrXII and chrXV) contain the largest number of peaks, showing a general good correlation between peaks’ distribution and chromosome content (see Table 1 in S1 File). However, peaks do not follow a regular pattern but show regions of intense presence as well as empty regions; the different distances between peaks are reported in Fig. 1 (inset).

The chromosomal map suggests that peaks may be positioned at some specific target sites. First, we compared peaks’ location to ORFs; then, to major genomic annotations. The results, reported S1 Table, show that most of flexibility peaks (170 peaks out of 183, 92.9%) are positioned within interORF regions (Fisher test: *p* < 10^−16^). Out of the remaining peaks, 11 lie inside ORFs, one peak lies on a telomere (peakI-2) and one peak lies on a rRNA locus (peakXII-12).

### Flexibility peaks are localized at tandem repeats inside 3′UTR regions

In *S. cerevisiae* compact genome the interORF regions make up only 27% of the genome length. Of them, 26% are upstream of two divergently transcribed genes and 49% are upstream of one gene and downstream of another, so including putative promoters; finally, 25% are downstream of two convergently transcribed genes, presumably containing only terminators [16].

The inspection of the interORF regions containing flexibility peaks reveals that 67 peaks (39, 4%) lie at interORF regions between converging genes, 77 peaks (45, 3%) lie between genes with unidirectional transcription, only 26 peaks (15, 3%) lie between two genes with divergent transcription (see S1 Table). This is not coherent with 1:2:1 ratio distribution of the yeast genome, making the difference statistically significant for the converging regions (Fisher test: *p* = 2, 959 × 10^−5^) as well as for the diverging ones (Fisher test: *p* = 2.201 × 10^−3^).

The distribution and position of genes along chromosomes are basic genomic features known to play a role in the regulation of gene transcription and translation; this is of particular importance in yeast compact genome due to its dense arrangement of genes and short intragenic regions. For example, genes that are divergently expressed may share promoter and transcription factors and show similar regulation and functional relationship; similarly, convergent genes may share terminators or 3′-transcribed regions [17]. In this context, the observed prevalent position of flexibility peaks suggests that they could represent structural regulatory signals.

We take advantage of measurement of promoter, 5′UTR, 3′UTR and terminator regions of a large number of yeast genes reported by Tuller et al. [17] to analyze the possible co-localization of any of these regions with flexibility peaks. According to the cited authors, promoters and terminators were considered the sequences intermediate between the different untraslated regions; for only a few ORFs without measure data, the average length of 5′UTR and 3′UTR were reported. We found that all peaks lying between convergent genes, except 4 peaks, co-localize with the 3′UTR of one ORF or of both ORFs, as in the cases of very large peak extension or 3′UTR partial overlap (Fisher test: *p* < 10^−15^). Peaks lying between genes with unidirectional transcription co-localize with 3′UTR in 64 cases (Fisher test: *p* < 10^−15^). To sum up, peaks on a 3′UTR region are 127 and ORFs with a peak in 3′UTR are 175. Finally, peaks between divergent genes co-localize with 5′UTR in 18 cases (Fisher test: *p* < 10^−15^). Peaks’ features are reported on S1 Table.

**Fig 2:**
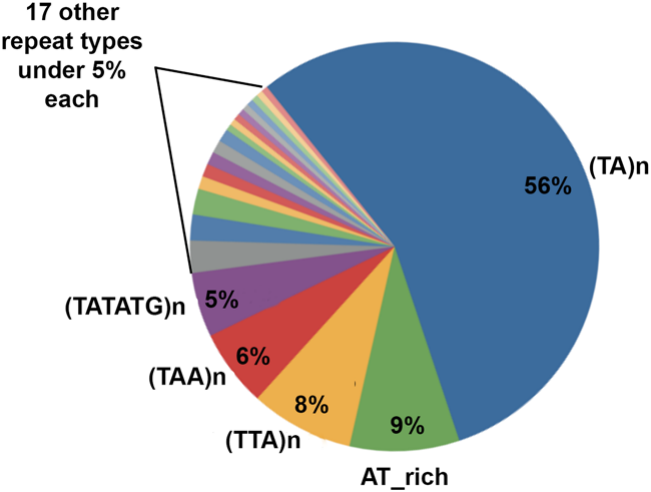
Distribution of repeats within flexibility peaks.

The presence of shared sequences inside peak sequences was searched by a ClustalW2 alignment analysis, that however give no significant results. Differently, a Repeat Masker analysis revealed that all peaks were characterized by (TA)n or similar AT-rich repeats (Fig. 2).

(TA)n repeats show a predominant presence and characterize all peak types except the 11 peaks lying inside ORFs, all of which contain (TTA)n. Repeats show a great length variability and comprise stretches of uninterrupted dinucleotide TA sequences mixed with degenerated TA sequences (from 23 to 89*bp*). For this reason, in the following we shall refer to all types of AT-rich sequences as to tandem repeats, indifferently.

### Flexibility peaks map on polyadenylation signals

3′UTR is a regulatory region; in yeast several distinct but interacting elements compose the 3′-end forming signals: the polyadenylation efficiency element (EE), the positioning element (PE) and the near-upstream/near-downstream elements (w.r.t. cleavage site). EE is the upstream signal including mainly TATATA (consensus sequence: TAYRTA). PE occurs 16 to 27*nt* downstream and the best word for this element is AATAAA (consensus sequence: AAWAAA); however, it is commonly described only as A-rich, since many functional sequences are characterized only by their adenosine content. The near-upstream element, as well as the near-downstream, is characterized as T-rich [18].

The EE promotes the recruitment of other polyadenylation factors by binding, upon transcription of RNA, the trans-acting factor *Hrp1*, that also plays important roles in mRNA export, mRNA surveillance and nonsense mediated decay. The TAYRTA sequence provides the greatest effect on 3′-end processing with the T/U at the first and fifth positions being the most critical for function; on a large-scale analysis (1017 yeast nuclear transcripts) more than half of 3′UTR (52%) contained this optimal EE sequence [19]; in more cases, transcripts contain several consecutive copies of EE sequence [20]. Owing to these reported TA-rich EE structures, we searched evidence for a general relationship between the tandem repeats (corresponding to flexibility peaks) and EE elements.

In literature, the sequence for the 3′-end of the *GAL7* or *MRP2* genes have been made available [20] and authors mapped in detail major poly(A) sites and expanded EE elements (TA)_8_. We found that the EE elements co-localize with an under-threshold flexible region (i.e. a genomic region where flexibility is enhanced, but does not reach the peak threshold). Similar results have been obtained for the expanded EE element detected within the 3′UTR of *FBP1* gene, constituted by a (TA)_14_ repeat [21], again co-localizing with an under-threshold flexible region; this last element is of special interest because it has been experimentally shown to be a very potent polyadenylation element in both strand orientations. The expanded EE has been suggested [22] to affect polyadenylation offering several overlapping binding sites to *Hrp1* or allowing its association/disassociation at multiple binding sites. Thus, we speculated that all the flexibility peaks that are positioned at 3′UTR might have the potential to serve as EEs, with an expansion linked to functional features, where the determinant for complex 3′-end formation could be just the DNA/RNA secondary structure due to helix flexibility.

Ozsolak et al. [23] have obtained very informative data in a map of poly(A) cleavage sites in yeast genome generated by a direct RNA sequencing.

**Fig 3:**
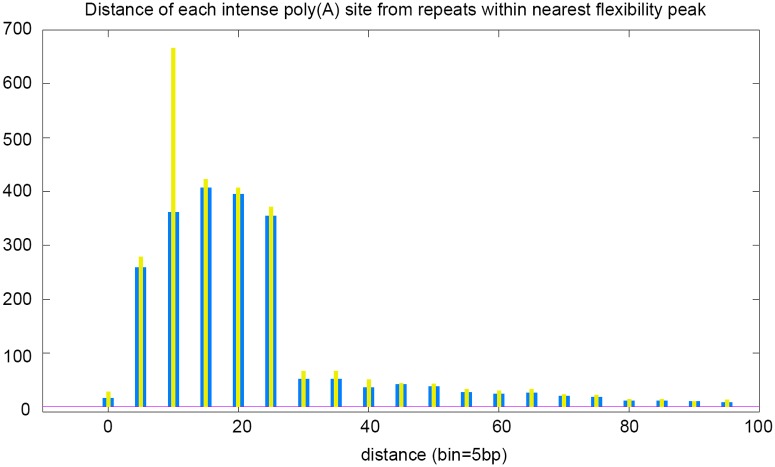
Distance of most intense poly(A) sites (score greater than 945)—following Ozsolak et al. [23]—from the midpoint of repeats inside each flexibility peak (see text for details on calculations). The outer bar (large and blue) refers to distance from (TA)n, only. The inner bar (thin and yellow) refers to distance from any repeat, indifferently.

For each poly(A) intense cleavage site (i.e. scored at least 945 by authors of [23]), we calculated the distance from midpoint of repeats in nearest peak. There are 2874 intense sites (out of 34444) which are closer than 500*nt* from a repeat within a peak. As shown by Fig. 3, intense poly(A) sites occur in a highly position-specific manner, prevalently within a distance range of 5*nt* to 25*nt* from repeats: 91.7% of them are closer than 100*nt* and 73.8% are closer than 25*nt*. If we limit this analysis only to (TA)n, then 75% are closer than 25*nt*. Poly(A) intense cleavage sites usually are present as multiple and clustered elements inside range [0-25*nt*] from repeats. Almost all peaks in convergent and unidirectional intergenic regions match to intense poly(A) signals. The authors of [23] read weak and isolated signals as indicative of a low transcriptional activity; this occurs only in nine peaks, so it is nearly negligble.

Moreover, we inserted on UCSC Genome Browser the position of characterized positioning elements (PE, whose consensus sequence is AAWAAA) and of efficiency elements (EE, whose consensus sequence is TAYRTA), defined for both strands through the Yeast Genome Pattern Matching [24]. The analysis of repeats position and of strand direction of signals highlights a peculiar organization of 3′UTR extremity or of its extension. In unidirectional intergenic regions, the repeat sequence covers the extremity of mapped 3′UTR or lies slightly outside it, bordering the downstream poly(A) signals; the EE element is found in multiple copies, all overlapping the repeat sequences. The PE element, when present, may be positioned either upstream the EE (within the 3′UTR), or downstream the complete 3′-end forming signal, as well as in both positions within the same 3′UTR. Examples include the 3′-ends of genes *IME1* (peakX-5), *DBF4* (peakIV-14) or *CDC53* (peakIV-5) (see supporting S1 file, figure 1).

**Fig 4:**
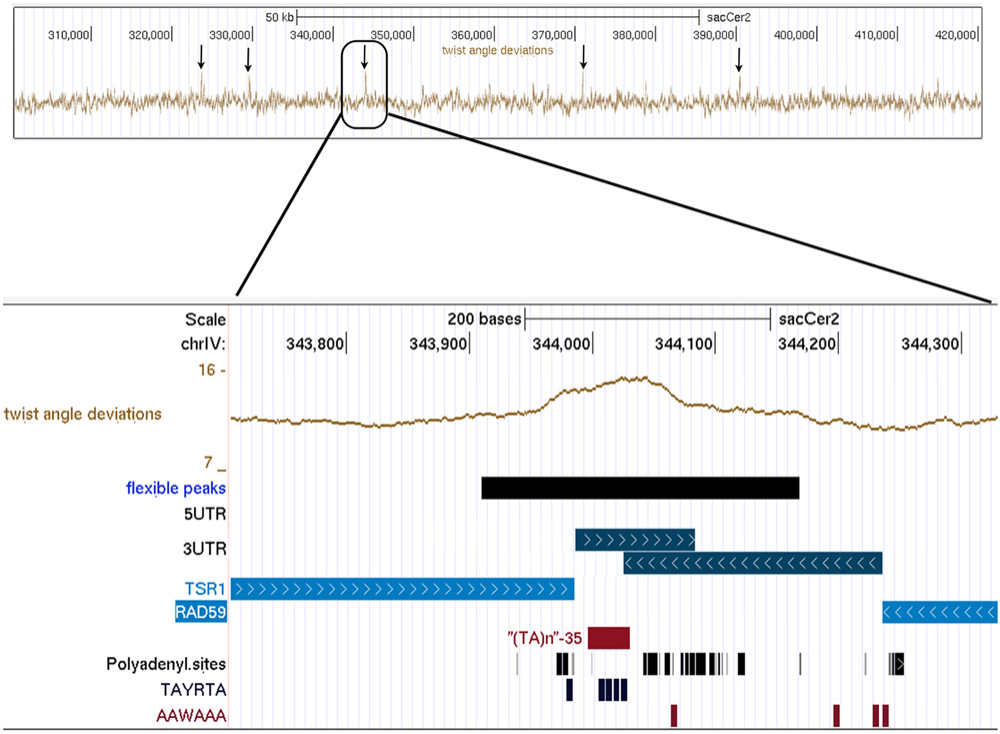
Snapshot of UCSC genome browser visualization of flexibility data on chrIV:300000-420000 region. Arrows target flexibility peaks. The bottom plot shows details for peakIV-9, lying within the convergent intergenic region between *RAD59* (*YDL059C*) and *TSR1* (*YDL060W*). Tracks correspond (in order from top to bottom) to Chromosomal location, Twist angle deviation values, Flexible peaks extent (values higher than 13.8*deg*), 5′ UTR and 3′UTR positions (5′UTRs are absent in this region, 3′UTRs are convergent), Annotated ORFs, (TA) Repeats from Repeat Masker, Polyadenylation cleavage sites from Ozsolak et al. [23], Polyadenylation signals (Efficiency elements with consensus sequence TAYRTA and Positioning Element with consensus sequence AAWAAA) from Yeast Genome Pattern Matching [24].

In the convergent intergenic regions, ORFs often overlap their 3′UTR; here, the repeat sequence and the concomitant EE element may lie either inside only one or inside both 3′UTRs, thus bordering poly(A) signals on both sides; the repeat/EE sequence represents a central element from which the poly(A) reads depart in divergent direction, forming a complex overlapping polyadenylation signal. Examples are the peculiar 3′-ends of the convergent gene pairs *TSR1* and *RAD59* (peakIV-9, see Fig. 4), as well as *ERV15* and *AME1* (peakII-10), *SNC1* and *MYO4* (peakI-1), or *DIG2* and *PHO8* (peakIV-27) (see supporting S1 file, figure 2).

Interestingly, also in most divergent intergenic regions we found very clear poly(A) signals inserted into to the typical organization repeat/EE/poly(A) previously described for 3′-ends; due to lack of 3′-ends in these regions, this is unexpected. Sometimes the 3′-end signals lie on 5′UTR with sense or antisense orientation as respect to the adjacent ORF, as it happens for the region within the divergent *PUF3* and *YEH1* genes (peakXII-3); in other cases signals are distant from ORFs without any overlap with its components, as for region of peakX-3 within the divergent *TDH2* and *MET3* genes (see supporting S1 file, figure 3). These findings clearly indicate the presence of termination signals in absence of annotated transcriptional units; therefore, peaks which are positioned at 3′UTR may also mark non coding RNA genes, that frequently may be antisense transcripts. A large quantity of antisense transcripts has been reported by both Ozsolak and Nagalakshmi studies [23, 25] and they are estimated to cover in yeast the 80% of annotated ORFs. Antisense transcripts are in lower amount and so are characterized by a low number of 3^′^-end signals; this motivates the presence of weak signals in peaks which are not positioned at 3′-end of ORFs.

Finally, concerning peaks lying inside an ORF, we remark that we found poly(AAT) codons coding for poly-*Asn* region of polypeptide—instead of poly(A) signals.

**Fig 5:**
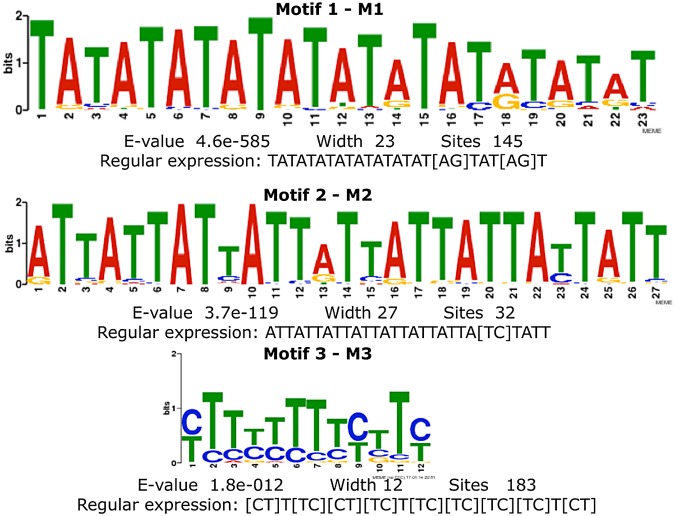
Significantly recurrent motifs identified by MEME algorithm [26] on peak regions. Motif 1 has the consensus sequence TATATATATATATATATGTATAT (E-value = 4.6 × 10^−585^) and is found in 145 peaks; motif 2 has the consensus sequence ATTATTATTATTATTATTATTATTATT (E-value = 3.7 × 10^−119^) and is found in 32 peaks. Motif 3 has the consensus sequence CTTCTTTTCTTC (E-value = 1.8 × 10^−12^) and is found in 183 peaks; in this case the analysis has been performed on peak sequence comprehensive of additional 100*nt* upstream and downstream.

On conclusion, TAYRTA elements, closely adjacent to cleavage site, have a non-canonical position in the peak-associated 3′UTRs. To explore the concomitant occurrence of further polyadenylation elements we performed a search for motifs by a MEME analysis [26], carried out on 183 peak regions. We identified, as expected, a TATATATATATATATATGTATAT motif (MEME statistical significance E-value = 4.6 × 10^−585^) in 145 peaks and a ATTATTATTATTATTATTATTATTATT motif (MEME statistical significance E-value = 3.7 × 10^−119^) in 32 of them. Moreover, performing an analogous analysis on flexible regions±100 (i.e. peak regions, comprehensive of additional 100*nt* upstream and downstream), we found that in 183 sites the novel A/T-rich motif CTTCTTTTCTTC (MEME statistical significance E-value = 1.8 × 10^−12^) was found (see summarizing Fig. 5). This last motif seems to have some function since it again occurs in all interORF peak regions.

Overlapping 3′UTRs are common in many genomes for genes orientated in a tail-to-tail manner. They have been described in yeast, where they may depend on the dense arrangement of genes and possibly to cause transcriptional interference [27]. It is credible that, similarly, for unidirectional genes, failure to terminate transcription at the end of first gene will result in inhibition of the next gene [28] and that this interference type could act as a regulatory system for the differential expression of adjacent gene pairs or for the sense-antisense transcription [29]. This suggests that the flexible elements inside 3′UTR could characterize genes with specific types of termination, where peculiar signals are required possibly to regulate a programmed RNA interference.

### Flexibility peaks are conserved and identify genes with decreased mRNA stability

Following the rationale that functional elements show a relative evolutionary conservation, we determined the conservation rate of flexible sequences in four other sequenced *Saccharomyces sensu stricto* species (*S. bayanus, S. paradoxus, S. mikatae, S. kudriavzevii*). For this analysis a dataset by Scannell et al. [30, 31], containing the alignment of 4298 intergenic regions, was analysed. Out of the 170 flexible sequences (excluding those inside ORFs), 131 regions (77%) conserve a flexibility peak exceeding the fixed threshold in at least one species and 70 regions (41%) in all species; in most cases of conservation failure, under-threshold flexible regions were observed. Conservation of peaks is particularly strong for the convergent and unidirectional intergenic regions. Out of the 67 convergent ones, 55 regions (82, 1%) conserve the flexibility peak in at least one species and precisely 53 in *S. paradoxus*, 52 in *S. mikatae*, 50 in *S. kudriavzevii* and 49 in *S. bayanus* (see S2 Table). Consistently, 51 out of the 55 conserved flexible sequences are in regions with conserved synteny maintaining convergent transcription. The unidirectional regions conserving a flexibility peak in at least one species are 67 (81, 8%), all maintaining unidirectional transcription. Differently, the peak conservation in divergent intergenic regions is significantly under-represented (50%; Fisher test: *p* = 0.002).

Of interest, the sequence alignments may show that conservation of peaks does not derive from the identity of intergenic sequence but is frequently consequent to a different organization of a high number of tandem repeats, as visible in the alignments of intergenic regions of peakIV-14 -unidirectional intergenic region between *DBF4* and *DET1*- and peakIV-9 -convergent intergenic region between *RAD59* and *TSR1* (see supporting S1 file, figure 4 and figure 5). These findings are indicative of an evolutive differentiation among species with a substantial conservation of flexibility peaks, even when there is a weak sequence conservation among the four genomes. Notably, 38 conserved flexibile ORFs (22 in converging and 11 in unidirectional transcription) were found to belong to the list of ohnologs i.e. paralogous genes arising from whole genome duplication [32] (see S2 Table); in all cases, except one, only one member of ohnolog pair carries a flexibility peak in 3′UTR. Usually, the pair members of ohnologs underwent sequence modifications related to functional changes of different extent. Consequently, the peak sequence on one onholog may be a peculiar modification linked to functional divergence between pair members, possibly leading to sub- or neo-functionalization, which are processes already defined in yeast for a number of duplicated genes [33].

The gene order arrangement has an evolutionary meaning [34]. In yeast, for instance, adjacent genes are co-expressed to a significantly higher level than expected [35]; moreover, many highly co-expressed gene pairs take part in the same cellular processes [36]. Accordingly, the conservation of flexibility peaks in convergent or unidirectional pattern may be related to the peculiar structural or functional aspects of gene pairs expression.

**Fig 6:**
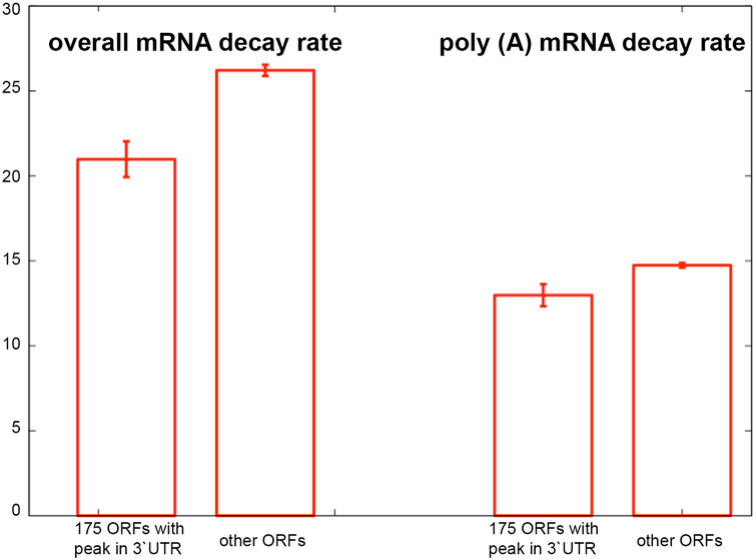
Comparison between overall mRNA decay rates (left) and poly(A) mRNA decay rates (right) in the 175 ORFs containing a 3′UTR peak against all the other ORFs (data from [38]). For each group, the histogram shows the mean value ± standard error of the half-lives of mRNAs -either overall or poly(A). The half-lives are measured in minutes.

The 3′UTR regulates mRNA levels or stability via RNA-protein interactions with mRNA degradation machinery. mRNA stability is a key regulatory step controlling gene expression and ultimately affects protein levels and function. Notably, long- and short-lived transcripts appear to have systematic differences in the EE, suggesting peculiar roles of this poly(A) signal in mRNA stability [37]. Therefore we checked whether the ORFs with peak in 3′UTR could be related with a differential mRNA stability. We took advantage of data about mRNA half-lives derived by Wang et al. [38] coming from mRNA decay profiles measured by microarrays following transcriptional shut-off. Results were searched for the 175 ORFs with peak in 3′UTR compared with all other ORFs; they show that these ORFs are characterized by significant lowering of both poly(A) half-life (*t*-test: *p* < 2.5 × 10^−2^) and overall half-life (*t*-test: *p* < 1 × 10^−2^), indicating their production of unstable mRNAs (see Fig. 6). According to current models for major decay pathways, in yeast poly(A) shortening precedes the decay of the entire transcript and is a rate-limiting step [39]. Differential degradation of mRNAs can play an important role in setting the basal level of mRNA expression and how that mRNA level is modulated by environmental stimuli. It has been suggested that there is a general relationship between the stability of an mRNA and the physiological function of its product. Accordingly, mRNAs involved in central metabolic functions are generally relatively long-lived, whereas those involved in regulatory systems turn over relatively rapidly [38]. Consistently, flexibility peaks inside 3′UTR may be proposed to be part of the regulatory machinery of short-lived mRNAs.

### Insights into the functions of ORFs with peak in 3′UTR

The prevalent occurrence of unstable transcripts for ORFs with peak in 3′UTR has obvious implications for their possible regulatory roles within specific pathways. A functional analysis of all such 175 ORFs (listed in S3 Table) was carried out by identifying the Gene Ontology (GO) terms, using the YeastMine search engine [40]. The search reveals enrichment for 72 GO Biological Process (*p* < 1.1 × 10^−2^) as well as for 14 GO Molecular Function categories (*p* < 2.6 × 10^−2^), as reported in S3 Table. The first 10 GO BP terms (i.e. with lowest p-value) are identified for a range of 31 to 86 ORFs per GO term, with a mean value of 62.3 ORFs per GO term. The GO MF term “binding” is identified for 101 ORFs.

Many GO terms concerned correlated processes or functions; so, they were processed by the web server REVIGO [41], using the default settings, in order to reduce their redundancy and summarize them in representative subsets the GO lists. The outcomes for Biological Process GO terms (visualized as treemap in supporting S1 file, figure 6, top) point out the presence of ORFs with role in cell cycle, phosphorus/organic cyclic compound/ nitrogen compound metabolism, phosphorylation reproduction, growth, response to acid, signaling. The 175 ORFs include genes expressing key components of cell cycle progression and regulation: *TUB2* and *TUB3* encoding *α* and *β* tubulins, *CLB4* and *PHO80* encoding cyclins, *CDC53* and *APC9* encoding respectively the cullin structural protein of SCF complexes and a subunit of the Anaphase-Promoting Complex/Cyclosome; moreover, *AME1*, *RAD24*, *RAD59* and *SWE1* involved in checkpoint maintenance, the *FUS3*, *DIG2* and *SLT2* encoding MAP-kinases and their regulator *BMH1* encoding the major isoform of 14-3-3 proteins. Further *IME1*, encoding a master regulator of meiosis and its convergent gene *UME6*, the key transcriptional regulator of early meiotic genes; moreover *MFA1*, encoding the essential mating pheromone a-factor, *STE50* the major protein involved in mating response. Finally, *ASG1*, *TSR1*, *ICT1*, *YAP1*, *PHO80*, *FRT1* and *HAA1*, regulators involved in the stress response. In accordance with the prevalent regulatory functions revealed for Biological Process GO terms, the REVIGO outcomes for Molecular Function GO terms point out the presence of numerous ORFs with role in binding and in phosphatase and kinase activities (visualized as treemap in supporting S1 file, figure 6, bottom).

All these findings confirm the general involvement of ORFs with peak in 3′UTR in regulatory systems as well as their characterization by unstable transcripts. Moreover, these results seem to be coherent with the picture where regulatory function of genes is related to short half-life [38].

In budding yeast, the ability of genes to respond to environmental changes has been related to nucleosome occupancy in 5′-ends and 3′- ends [42, 43]. Nucleosome free regions or nucleosome depleted regions (NFR or NDR) were observed at regulatory regions such as gene TSS and TTS, affecting binding of regulatory proteins, nucleosome ordering inside genes and transcriptional plasticity [44, 45]. Since AT-rich sequences in defined contests have nucleosome-disfavoring property, we evaluated whether the AT-rich sequence in flexible peaks in 3′UTR could play a regulatory role by determining specific nucleosome positioning; thus, we analyzed the co-localization of peaks with NDR, obtained from [46]. We found that large distances occur between each peak and nearest segment with high nucleosome depletion (Fig. 7 in supporting S1 file), indicating that AT-rich peak regions and NDR are not associated elements. A manual inspection was then performed on nucleosome occupancy of all peaks localized in 3′UTR of convergent genes, to be sure to consider only transcriptional terminators. Data on experimental nucleosome occupancy, reported by [47], together with nucleosome coverage predicted by a model based on *in vitro* sequence data, were available through the SwissRegulon server [48, 49]. We found that no peak shows altered nucleosome coverage. These are unexpected results, as many papers describe nucleosome depletion in yeast gene 3′-end termination. Anyway, they contribute to circumstantiate the flexibility peak’s action, by suggesting that flexible peak may exert exert its function on polyadenylation by affecting phases not directly dependent on local chromatin structure, for example by modulating the nascent mRNA structure.

**Fig 7:**
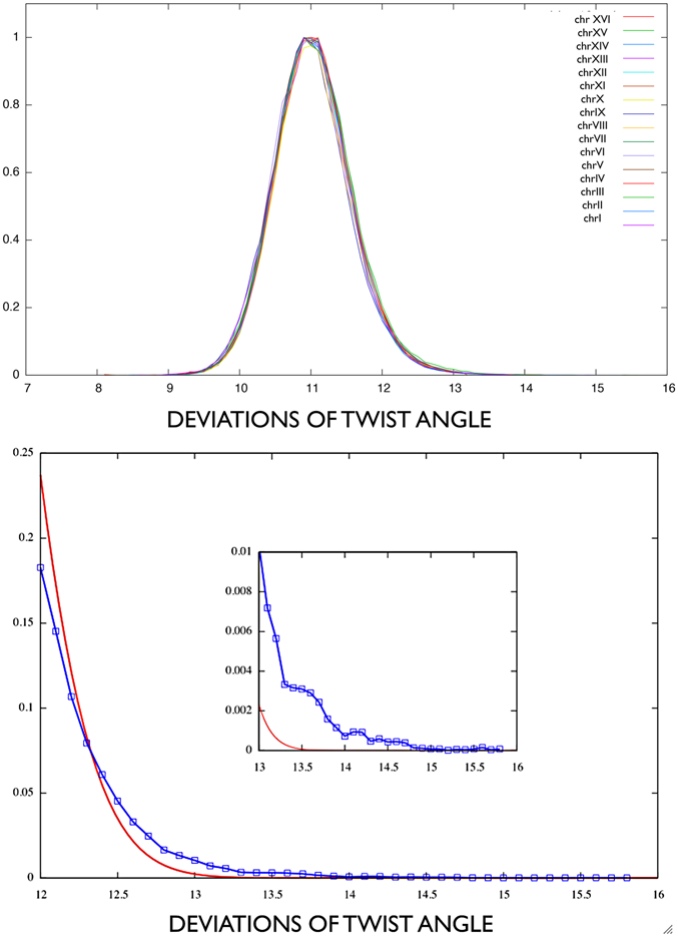
A: Flexibility values normalized distribution for all the yeast chromosomes. B: Upmost tail for flexibility values greater than 12*deg* (within chrXI), compared to a Gaussian distribution with same mean and standard deviation. In-plot: values greater than 13*deg*.

Considering the gene function of peak associated ORFs, it is of interest that 14 of such ORFs have human orthologs involved in Mendelian diseases, detected from the Database of Human Disease Orthologs [50]; among these are the *YPL164C* gene, whose human ortholog gene *MLH3* encodes the DNA Mismatch Repair Protein *Mlh32* associated to HNPCC or Hereditary nonpolyposis colorectal cancer, the *YOL071W* (*SDH5*) gene whose human ortholog *SDHAF2* (alias *PGL2*) is associated to familial paragangliomas 2 and the YPL204W gene whose human ortholog CSNK1A1 is associated to familial adenomatous polyposis. A complete description of the human ortholog genes related to diseases is reported in S4 Table, including, besides genes, related diseases and detailed references, the chromosome band localization and the coincidental occurrence of common fragile sites. We highlight that the map position of the human ortholog genes for eleven yeast genes is coincidental with that of known fragile sites [51]; moreover most of orthologs are implied in cancer development. These findings support the relationship between peak associated ORFs and fragile sites.

We remark also the presence of *NIT3* among flexible yeast genes, a gene encoding one of two proteins that in *S. cerevisiae* have similarity to the mouse and human *Nit* protein, interacting with the human *Fhit* tumor suppressor. Indeed, the *FHIT* gene spans *FRA3B*, the most common human fragile site characterized for the presence of clusters of high flexibility peaks [52]. The *FHIT* gene has been suggested to have biological effects similar to *NIT* and to share with it signaling pathways [53].

## Discussion

In this paper we sistematically study the presence of flexibility peaks in *S. cerevisiae* genome and explore their functional role.

Peaks show a strong co-localization with tandem repeats inside the 3′UTR region of a number of ORFs and in particular with clusters of poly(A) signals. The peculiar architecture of repeats and poly(A) signals inside peaks suggests that they could mark terminations in ORFs characterized by specific requirements in RNA cleavage. Consistently, we characterize the peak presence in ORFs as prevalently lying in regions where convergent transcription occurs. Peaks show a general conservation among different *Saccharomyces* yeast species, but with a sequence variation in orthologous genes and a clear differentiation between paralogous genes, suggesting that they could be the result of an evolutive differentiation. We provide evidence that ORFs with peak in 3′UTR have transcripts with lower half-life, item considered peculiar of genes involved in regulatory systems with high turnover. More, we show that ORFs with peak in 3′UTR share a number of common functions in biological processes such as cell cycle regulation or stress response. From these findings we infer that flexibility peaks could play a functional role as regulatory elements of gene expression for a peculiar set of genes. A regulation based on flexible sequences has not so far experimental foundation. However, we must consider that, while the impact of 3′-end sequence on gene expression is well established, the understanding of how its effect is encoded in DNA is limited. Polyadenylation is critical for many aspects of mRNA metabolism, including mRNA sytability, translation and transport. PolyA signals act as substrate for cleavage and polyadenylation, for which RNA structure is also a critical determinant [54]. Then, RNA binding proteins regulate almost all post-transcriptional stages [55]. Specific sequence motifs in 3′UTR have been identified in yeast implied in stabilization [56] and stress response [57]. In particular, an increased AT-content upstream the polyadenylation site has been shown to modulate protein expression dynamics [58]. Thus, AT rich tandem repeats and strand flexibility may be crucial in determining the interaction with polyadenylation factors, the mRNA structure and the accessibility of binding sites to multiple regulators. The notion that enriched tandem repeats in *S. cerevisiae* could guide transcriptional modulation has been established for genes carrying very variable tracts of repeats in promoter; the involved genes have the general feature of interacting with the cell environment and so requiring rapid response changes [59, 60]. Gene regulation differs greatly among related species, constituting a major source of phenotypic diversity. This issue assumes relevant significance for gene evolution and tandem repeats have been considered able to drive transcriptional divergence and to confer evolvability to gene expression [61]. The variable repeat-based component of peaks inside 3′UTR may have similar origin and evolution. Tandem repeats are intrinsically prone to variation having often units lost or gained by replication slippage [62]: Thus, long repeat stretches could be derived from the well-known polyadenylation enhancement elements; their potential in modulating gene expression regulation (termination efficiency and transcript half-life) may have been the feature that determined their fixation in peculiar genes.

These findings on yeast genome may be relevant for the knowledge of the relationship between flexibility peaks and human genome instability. Common fragile sites are chromosome regions prone to breakage upon replication stress. To date, 22 fragile sites, among the 230 mapped in human lymphocytes, are known at molecular level but the molecular basis of fragility remains unknown. They extend over megabase-long regions, tend to overlap very large genes and share a delayed completion of DNA replication. Recently, delayed replication has been correlated with a paucity of initiation events [63, 64]. Notably, the authors found that FRA3B and FRA16D, the most active fragile sites in human lymphocytes, have low levels of fragility in fibroblasts, where instead other sites show very high fragility; cell-type-specific replication programs characterize the commitment to fragility at different loci in each cell-type, indicating that fragility is epigenetically defined.

These findings are consistent with the view that fragile sites serve a function; this is supported by a number of indirect but relevant observations, the first of which is the conservation of fragile sites in synteny regions in the mouse and human genomes in all cases analyzed so far. The second one is their enrichment in genes related to cell cycle regulation, apoptosis or similar processes involved in cancer development [65]. More in detail, chromosomal fragile sites FRA3B and FRA16D, carrying the FHIT and WWOX genes respectively, that are genes playing a major role in apoptosis, show correlated expression and association with failure of apoptosis in lymphocytes from cancer patients [66]. In the same perspective, all fragile sites belong to networks of correlated breakage, comparable to gene expression pathways activated in response to damage stress; in particular the correlated fragile sites, analyzed in lymphocytes, are enriched in genes involved in immunity and inflammation, that are cell-type specific processes of lymphocytes [8].

Coherently with the above described functional aspects, flexibility peaks in yeast occur in ORFs involved in cell cycle control or stress response, where flexible sequences seemed to play a regulatory role in gene expression. While yeast is a unicellular and quite simple organism, many processes are highly conserved; it is conceivable that conservation may concern the specific mechanisms that differentiate the expression of peculiar gene classes. In higher eukaryote evolution, these mechanisms may have been used in the commitment of the different genes to stress response, that is cell and tissue specific [67].

In this view, the regulatory role of flexibility peaks inferred for yeast genes could be actual also for human fragile genes, even if not necessarily involving 3′-end termination process. The extent of this correlation will be determined by a comparable genome-wide analysis on human sequence DNA flexibility.

## Materials and Methods

### Genomic data

We refer to complete *Saccharomyces cerevisiae* RefSeq genome as obtained and annotated on SGD (SacCer2 assembly).

### StabFlex algorithm

Experiments on conformational analyses of DNA require large numbers of conformations to be sampled. The conformation of DNA and its sequence dependence are mainly determined by the chemical structures of the base pairs and their interactions. The computational model by Sarai et al [68] examines DNA flexibility on the basis of base pairs interactions and the results agree with available experimental observations. The algorithm StabFlex is used to calculate potential local variations in the DNA structure that are expressed as fluctuations in the twist angle (degrees, *deg*). It is a reimplementation of the TwistFlex software [11] and it is targeted to analyze very large sequences.

### Flexibility values and peaks

The calculation of twist fluctuations is made for overlapping windows along a given sequence (window length *L* = 100*bp*, window shift *s* = 1*bp*). Within each window the flexibility is calculated for consecutive dinucleotide steps, and the average value of all steps in the window is assigned to the midpoint dinucleotide step. The flexibility is measured in degrees (*deg*) in the range [7 *deg*;16 *deg*].

An example of the output data is given in Fig. 4. Peaks emerge spontaneously as short genomic regions where signal is extremely high. They are marked by arrows in the top picture. The complete flexibility data for a genomic region are plotted as a quantized signal and each flexibility value refers to 100*bp*, as shown in the bottom zoomed snapshot.

Fig. 7 (top picture) shows the normalized distribution of windows flexibility values for all 16 chromosomes of yeast genome. As shown in Fig. 7 (bottom picture), for large flexibility values (greater than 12*deg*) the distribution is no longer Gaussian. The non-Gaussian tail identifies flexibility peaks, as follows. First, we pre-selected regions with outstanding flexibility values, deviating significantly from the average (not lower than 𝓢 = mean+2×stand dev, which is 12.1 for all chromosomes). That value 12.1 may be read as the point where Gaussianity is lost (see inplot in Fig. 7). Regions correspond to the genomic sequence covered by overlapping consecutive windows simultaneously exceeding 𝓢. Second, such regions whose maximal flexibility value exceeds threshold *θ* = 13.8 are defined flexibility peaks. The threshold has been fixed as in literature [12, 52].

Peaks have been denoted by peakIV-16, meaning the 16th peak within chrIV.

### Statistical analysis

The statistical significance of properties and classifications has been assessed by means of Fisher’s exact test and *t*-test. Fisher’s exact test is used in the analysis of 2 × 2 contingency tables built for categorical data that result from classifying objects in two different ways; it is used to examine the significance of the association (contingency) between the two kinds of classification. A *t*-test is a statistical hypothesis test in which the test statistic follows a Student’s *t* distribution if the null hypothesis is supported. It can be used to determine if two sets of data are significantly different from each other, and is most commonly applied when the test statistic would follow a normal distribution if the value of a scaling term in the test statistic were known. For both tests, specific R programs have been designed and implemented by the authors.

Differently, when external classifications have been used, statistical significance has been imported with the results. This applies to motifs found by MEME and to GO terms’ enrichment. As stated by the authors in [26], MEME usually finds the most statistically significant (low E-value) motifs first. The E-value of a motif is based on its log likelihood ratio, width, sites, the background letter frequencies, and the size of the training set. The E-value is an estimate of the expected number of motifs with the given log likelihood ratio, and with the same width and site count, that one would find in a similarly sized set of random sequences.

Concerning GO terms, as stated in [69], there are a number of different tools that provide enrichment capabilities. Tools differ in the algorithms they use, and the statistical tests they perform. All enrichment widgets list a term, a count and an associated p-value. The term can be something like a publication name or a GO term. The count is the number of times that term appears for objects in your list. The p-value is the probability that result occurs by chance, thus a lower p-value indicates greater enrichment without corrections. The p-value is calculated using the Hypergeometric distribution.

### Supporting information

A data repository for deviations of twist angle for complete yeast genome may be found in [13]. Individual chromosomal flexibility peaks’ annotations in BED format, suitable for a visualisation through the Genome Browser [15] are part of online supplementary material. The algorithm StabFlex is available at http://home.gna.org/stabflex/.

## Supporting Information

S1 File Peaks and ORFs involved. A.pdf file containing: a summary table on peaks and chromosome length; UCSC snapshots for peaks within unidirectional, convergent and divergent intergenic regions; alignments of peakIV-14 and peakIV-9 for Saccharomyces sensu stricto species; treemaps of the outcomes of REVIGO for Biological Process and Molecular Functions GO terms, referring to 175 ORFs characterized in 3′UTR by a peak; results of the comparison of peaks with the nucleosome depleted regions.
(PDF)

S1 Table Genomic features of peaks. A.csv table containing all genomic features corresponding to flexibility peaks.
(CSV)

S2 Table Conservation of peaks. A.xls file containing six tables about conservation in *Saccharomyces sensu stricto* species, ohnologs and synteny of ORFs involved in flexibility peaks.
(XLS)

S3 Table ORFs involved in peaks in their 3′UTR. A.xls file containing the list of 175 ORFs with peak in 3′UTR and tables about GO terms results.
(XLS)

S1 Archive Peak positions. An archive containing the flexibility peaks positions, in.bed format, suitable for UCSC visualization.
(ZIP)

S4 Table Human genes ortholog to ORFs involved in peaks. A.xls file containing the list of disease-associated human genes which are ortholog to yeast ORFs associated to peaks.
(XLS)
