# Supplementary material for: Global Mapping of DNA Conformational Flexibility on Saccharomyces cerevisiae
Source: PLoS Comput Biol. 2015 Apr 10;11(4):e1004136. doi: 10.1371/journal.pcbi.1004136 (PMC4393290; doi:10.1371/journal.pcbi.1004136)
Supplement: S1 File — A.pdf file containing: a summary table on peaks and chromosome length; UCSC snapshots for peaks within unidirectional, convergent and divergent intergenic regions; alignments of peakIV-14 and peakIV-9 for Saccharomyces sensu stricto species; treemaps of the outcomes of REVIGO for Biological Process and Molecular Functions GO terms, referring to 175 ORFs characterized in 3′UTR by a peak; results of the comparison of peaks with the nucleosome depleted regions. (PDF) [file pcbi.1004136.s001.pdf]

SUPPLEMENTARY PLOTS for paper Global mapping of DNA Conformational Flexibility on *Saccharomyces cerevisiae*

by Giulia Menconi, Andrea Bedini, Roberto Barale and Isabella Sbrana

This document contains plots and graphs which is referred to in the text of main paper.

Table 1: Summary on flexibility data for each yeast chromosome: length, measurements over threshold θ, regions and peaks. Finally a global view of genome covered by flexible zones is given.

Chrm	Length (bp)	Peaks
I	230208	2
II	813178	11
III	316617	7
IV	1531919	27
V	576869	12
VI	270148	5
VII	1090947	20
VIII	562643	11
IX	439885	4
X	745742	5
XI	666454	13
XII	1078175	19
XIII	924429	14
XIV	784333	5
XV	1091289	18
XVI	948062	10
	genome covered	0.38%

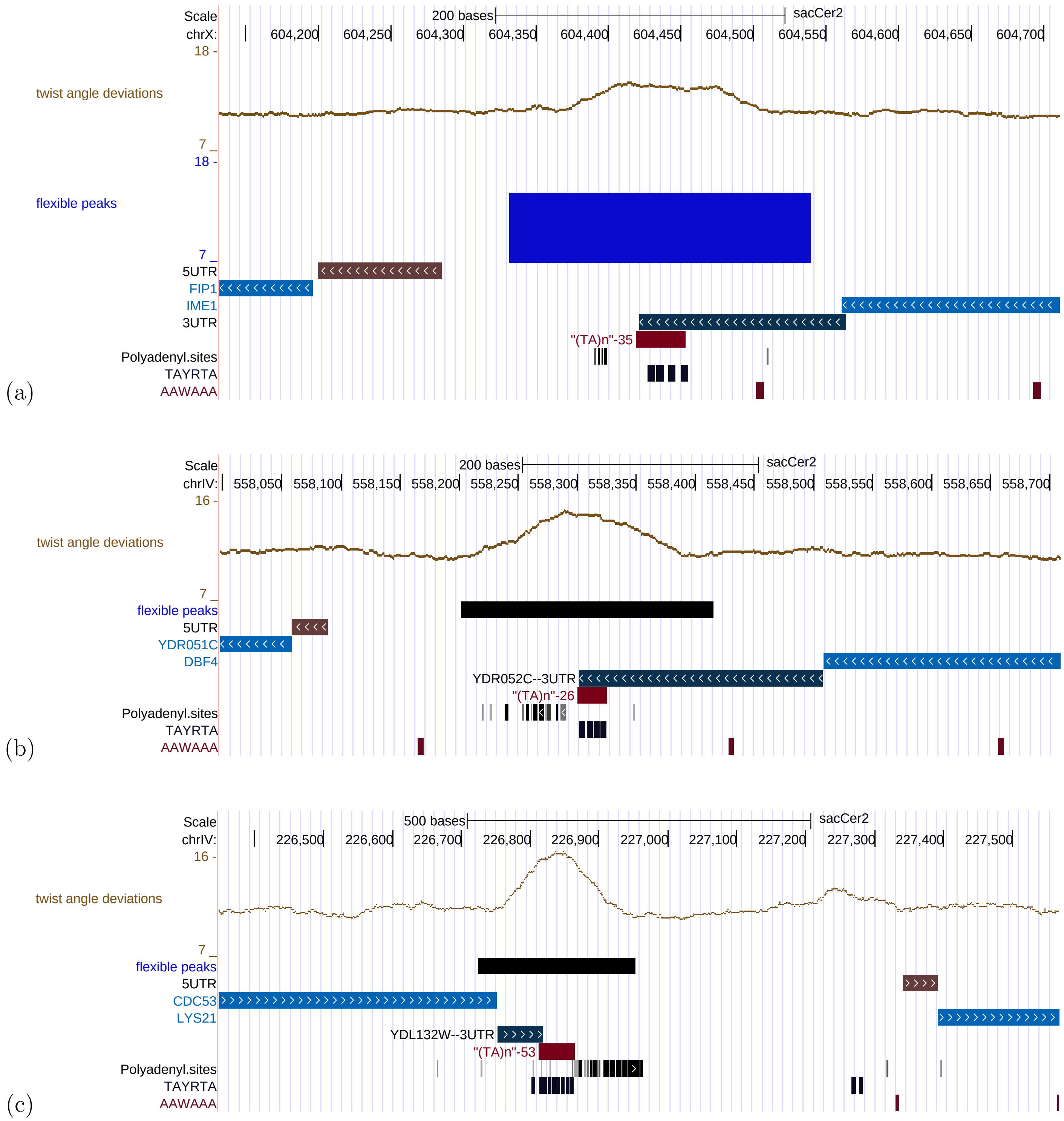

Figure 1: Snapshot of UCSC visualization of flexibility data for some peaks lying at 3′UTR unidirectional intergenic regions: (a) `peakX-5` between *IME1* (YJR094C) and *FIP1* (YJR093C); (b) `peakIV-14` between *DBF4* (YDR052C) and *DET1* (YDR051C); (c) `peakIV-5` between *CDC53* (YDL132W) and *LYS21* (YDL131W).

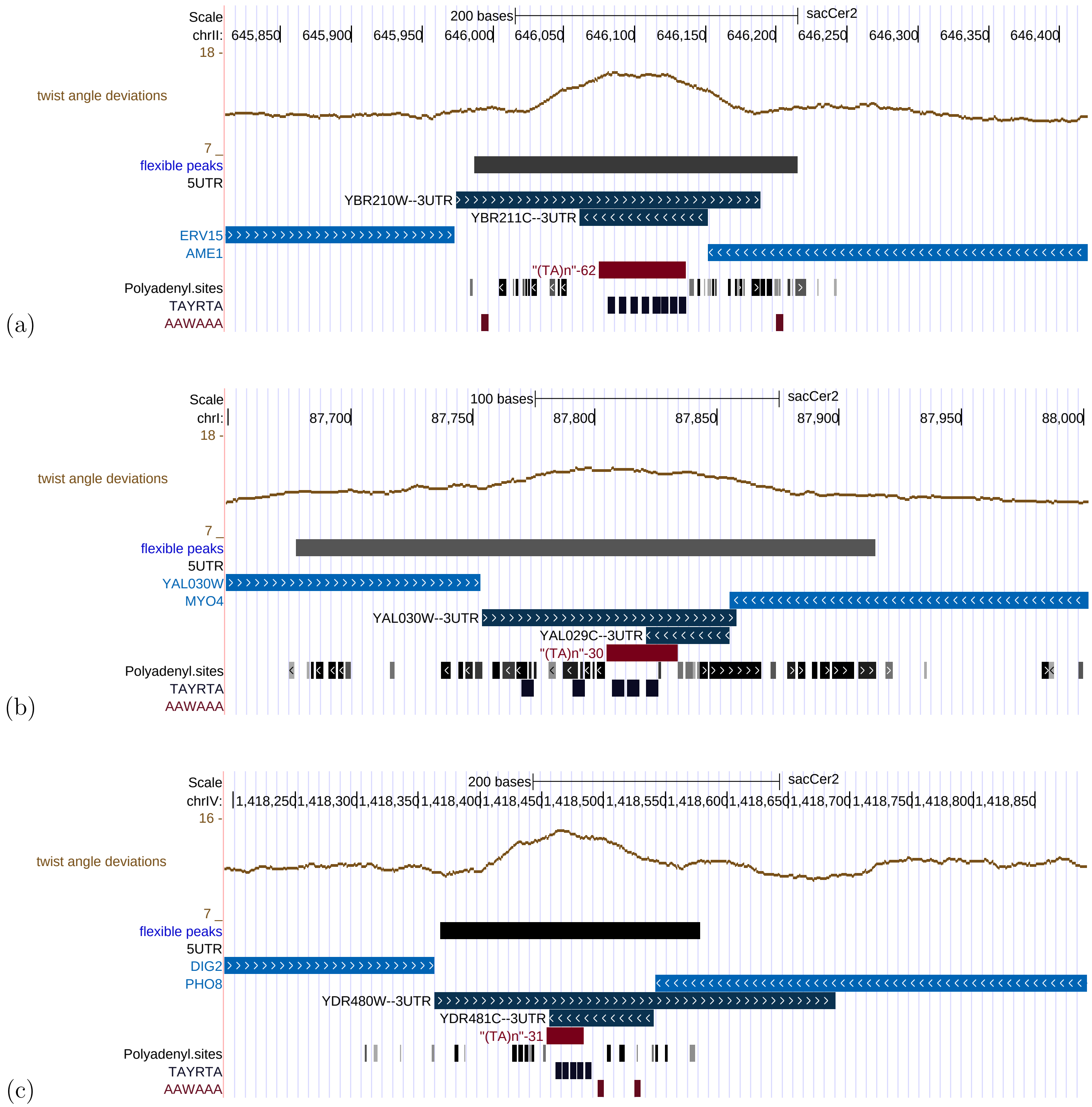

Figure 2: Snapshot of UCSC visualization of flexibility data for some peaks lying at convergent intergenic regions: (a) `peakII-10` between *ERV15* (YBR210W) and *AME1* (YBR211C); (b) `peakI-1` between *SNC1* (YAL030W) and *MYO4* (YAL029C); (c) `peakIV-27` between*DIG2* (YDR480W) and *PHO8* (YDR481C).

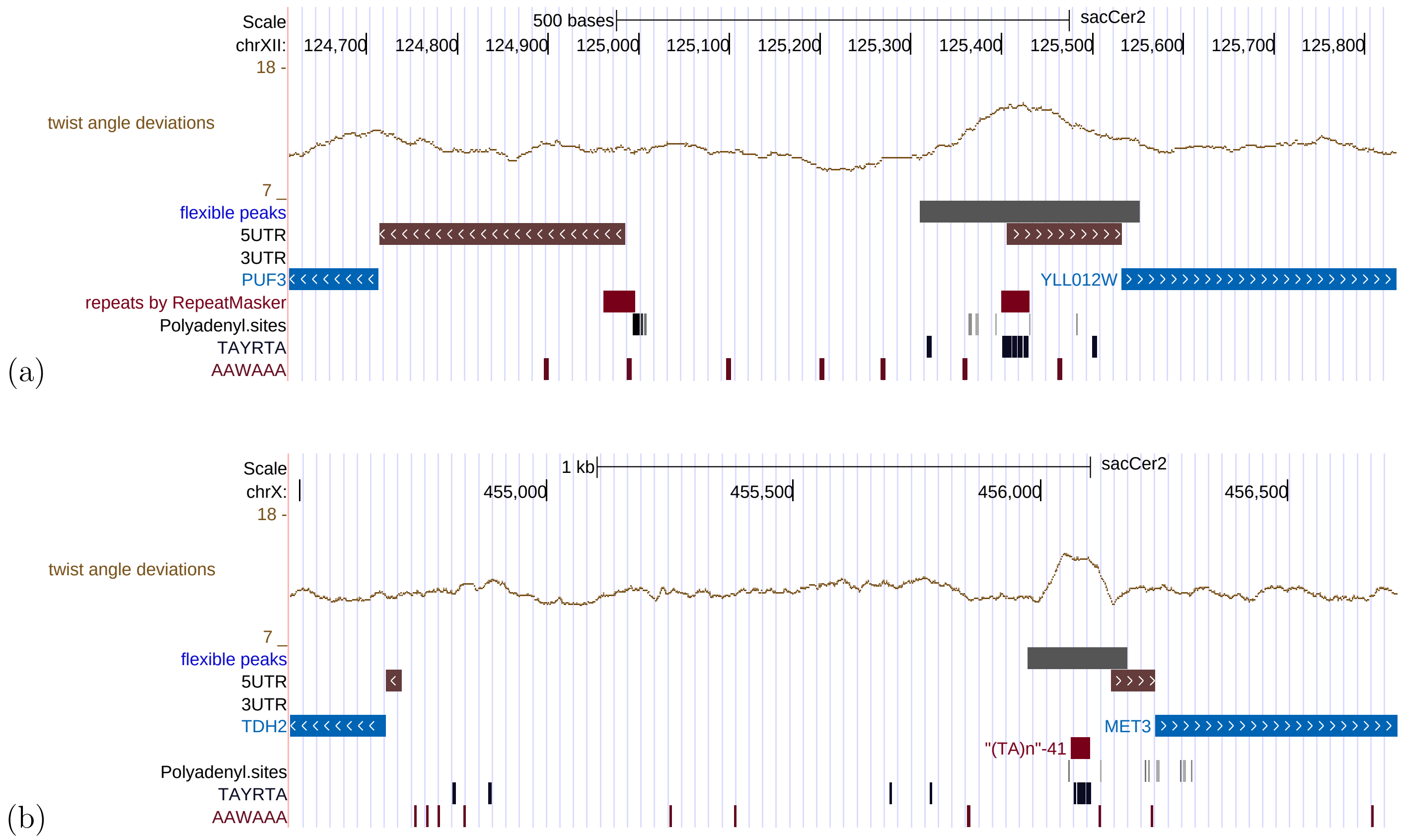

Figure 3: Snapshot of UCSC visualization of flexibility data for some peaks lying at divergent intergenic regions: (a) `peakXII-3` between *PUF3* (YLL013C) and *YEH1* (YLL012W); (b) `peakX-3` between *TDH2* (YJR009C) and *MET3* (YJR010W).

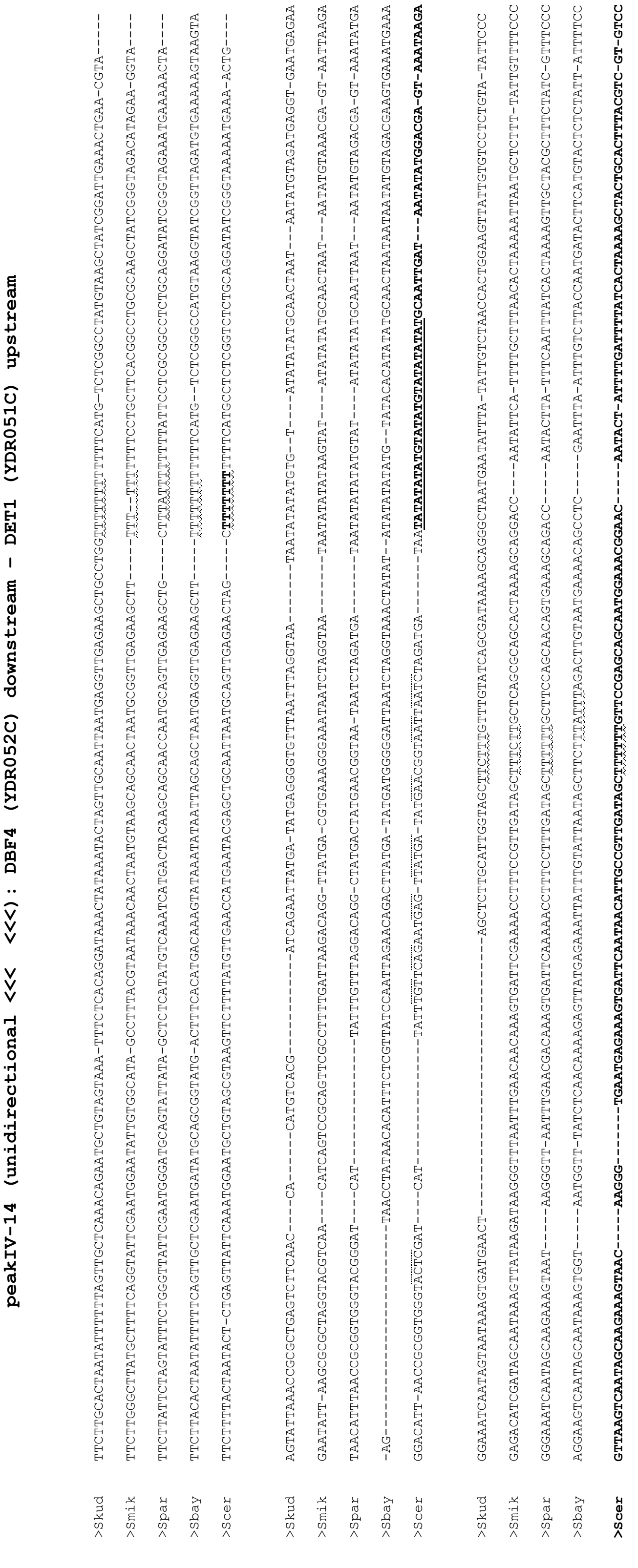

Figure 4: Alignment of *DBF4*-*DET1* unidirectional intergenic region for *Saccharomyces sensu stricto* species (`peakIV-14`). For each genomic sequence, motif AAWAAA is underlined and waved. Only for *S. cerevisiae*, TA repeats are underlined, poly(A) sites are overlined and 3′UTR is shown in bold.

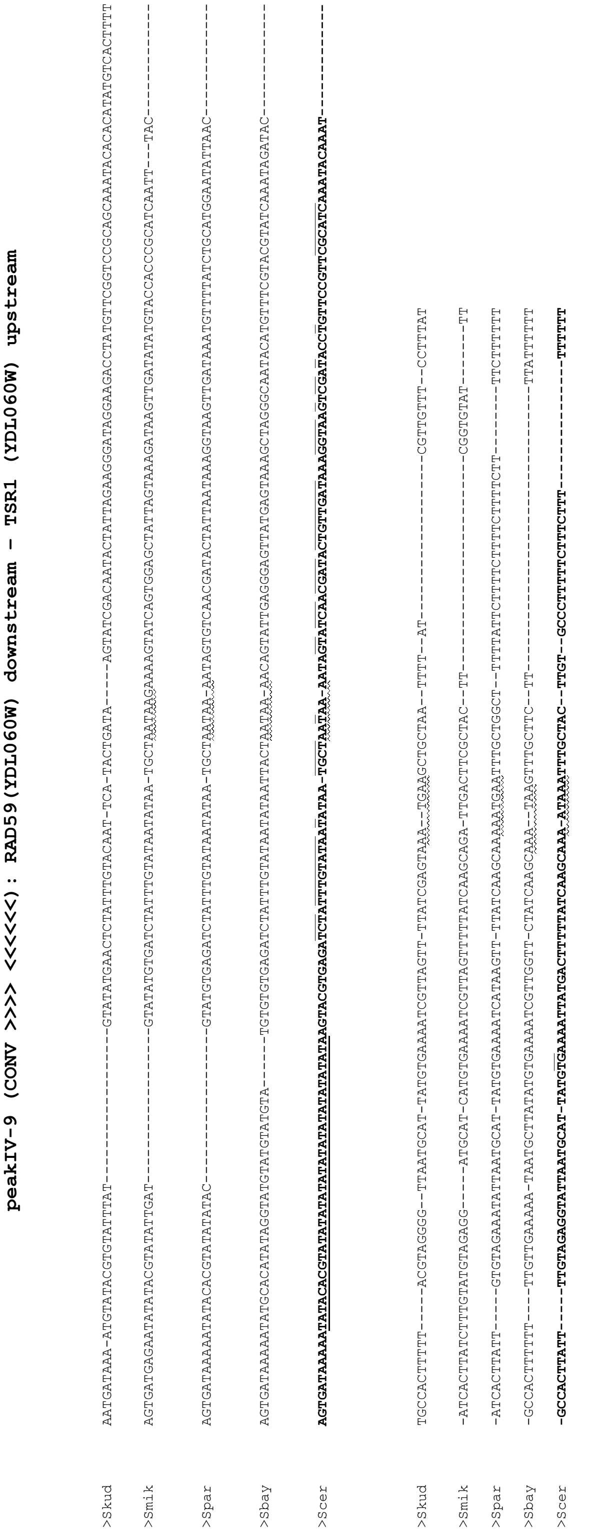

Figure 5: Alignment of *RAD59-TSR1* convergent intergenic region for *Saccharomyces sensu stricto* species. `peakIV-9` is on 3′UTR of *RAD59*. For each genomic sequence, motif AAWAAA is underlined and waved. Only for *S. cerevisiae*, TA repeats are underlined, poly(A) sites are overlined and 3′UTR is shown in bold. The peak is not conserved in *S. kudriavzevii*.

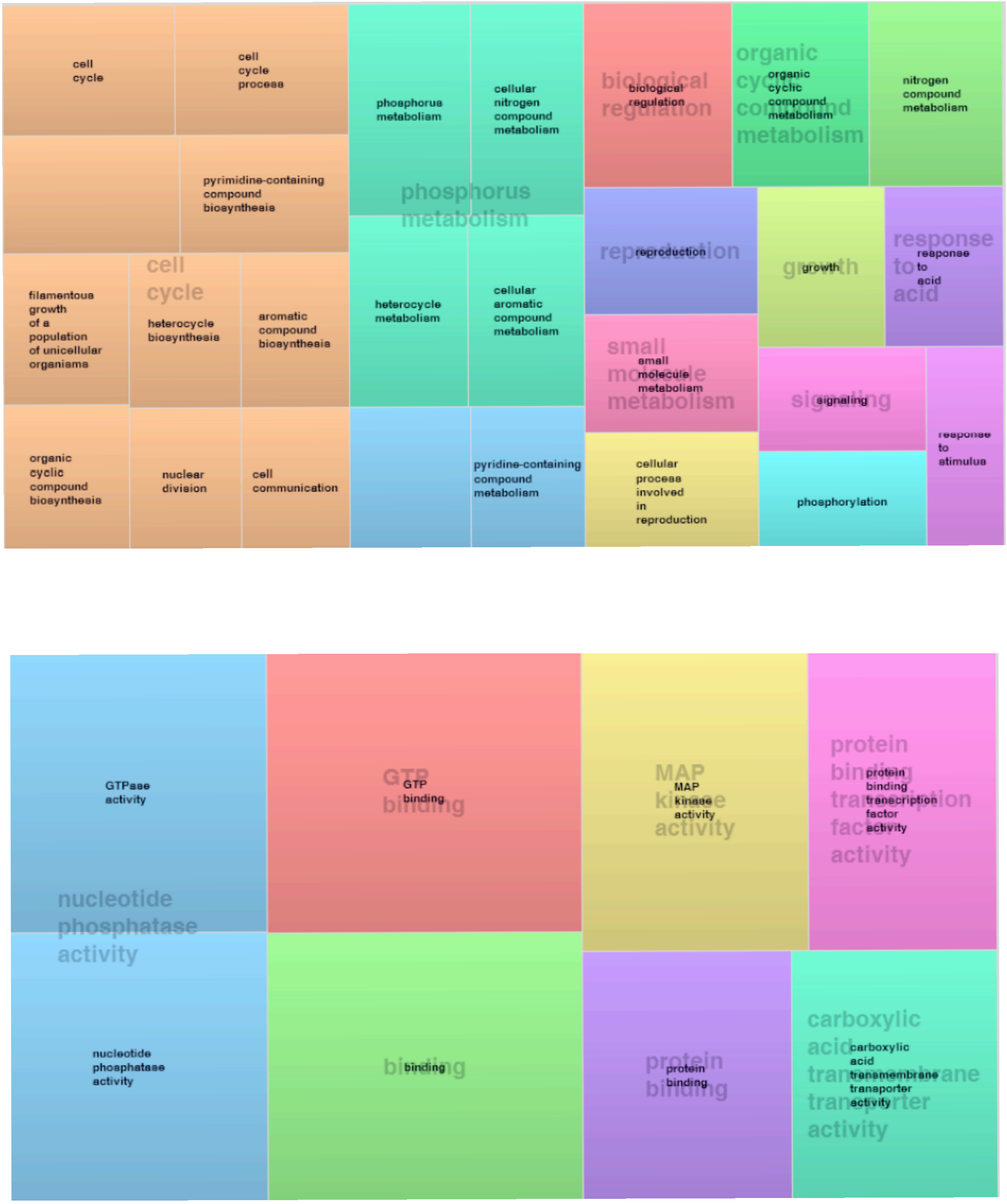

Figure 6: Treemaps of the outcomes of REVIGO for Biological Process (Top) and Molecular Functions (bottom) GO terms, referring to 175 ORFs characterized by a peak in 3′UTR .

Nucleosome depletion and flexibility peaks

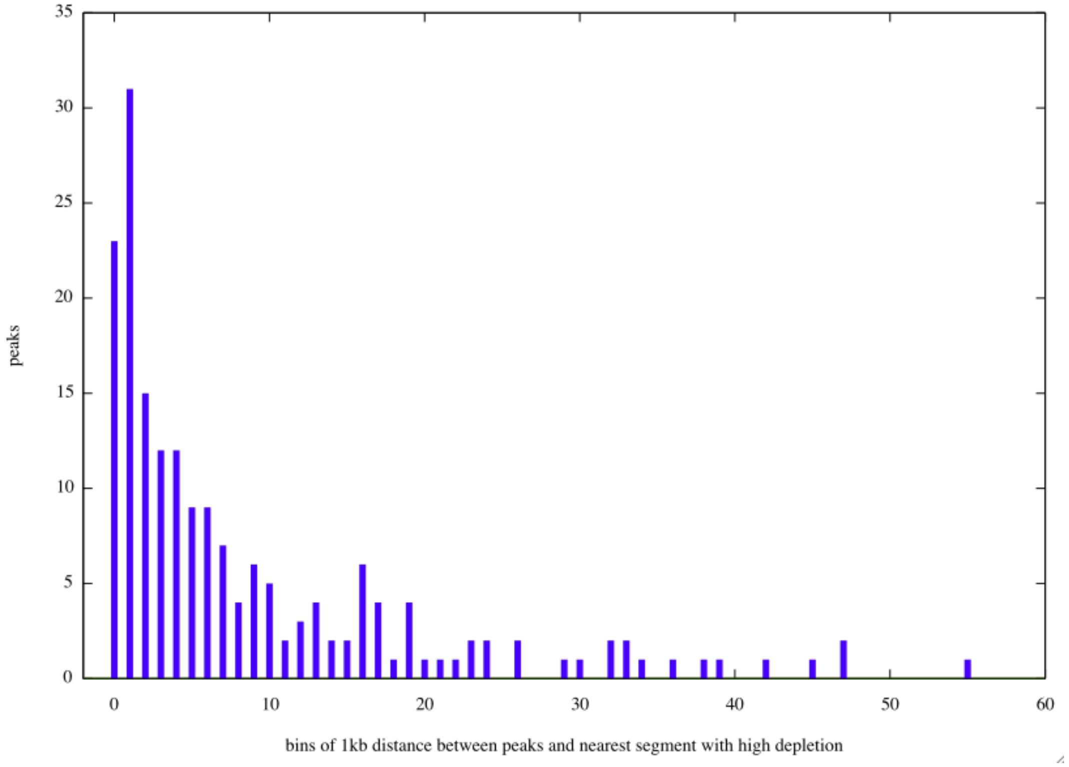

Figure 7: Distribution of distance of peaks from nearest genomic regions with strong nucleosome depletion (the so-called boundaries). Bins are 1kb and count for peaks within that distance from nearest boundary. Only 23 peaks are closer than 1000bp. Data are from: Field Y, Kaplan N, Fondufe-Mittendorf Y, Moore IK, Sharon E, et al. (2008) Distinct Modes of Regulation by Chromatin Encoded through Nucleosome Positioning Signals PLoS Comput Biol 4(11): e1000216.
