# Supplementary material for: Global Mapping of DNA Conformational Flexibility on Saccharomyces cerevisiae
Source: PLoS Comput Biol. 2015 Apr 10;11(4):e1004136. doi: 10.1371/journal.pcbi.1004136 (PMC4393290; doi:10.1371/journal.pcbi.1004136)
Supplement: S1 Archive — An archive containing the flexibility peaks positions, in.bed format, suitable for UCSC visualization. (ZIP) [file pcbi.1004136.s005.zip › readme_bedpeaks.rtf]

Each flexibility peak is indicated by its starting/ending position, together with the peak height.
